# Correction: Social distancing to slow the US COVID-19 epidemic: Longitudinal pretest–posttest comparison group study

**DOI:** 10.1371/journal.pmed.1003376

**Published:** 2020-10-06

**Authors:** Mark J. Siedner, Guy Harling, Zahra Reynolds, Rebecca F. Gilbert, Sebastien Haneuse, Atheendar S. Venkataramani, Alexander C. Tsai

After publication of this article, the authors were alerted by Katherine Baggaley of Popular Science to a possible error in the article. After subsequent investigation of the error she identified, and after consultation with a statistical reviewer, the authors identified additional errors.

**1. Incorrect calculation of doubling time noted by Baggaley**

In the second sentence of the second paragraph of Results, the text incorrectly reads: "At the date of implementation of the first social distancing measure, states had a mean daily case growth rate of 30.8% (95% CI 29.1–32.6; Table 1), corresponding to a doubling of total cases every 3.3 days." Baggaley correctly identified that the reported doubling time of 3.3 days did not accurately correspond to the reported mean daily case growth rate of 30.8%.

The corrected text should read: "Approximately one incubation period after implementation of the first social distancing measure, states had a mean daily case growth rate of 30.8% (95% CI 29.1–32.6; Table 1), corresponding to a doubling of total cases every 2.6 days."

In the second sentence of the first paragraph of Discussion, the text incorrectly reads: "Our estimates imply a more than doubling in the doubling time (from 3.8 days to 8.0 days) by 3 weeks following the implementation of social distancing measures." Baggaley correctly identified that the reported doubling time of 3.8 days was inconsistent with the doubling time described previously in the Results.

The corrected text should read: "Our estimates imply an approximate doubling of the doubling time (from 2.6 days to 5.2 days) by 3 weeks following the implementation of social distancing measures."

**2. Incorrect calculation of mean daily case growth rates and accompanying estimates**

While responding to Baggaley's queries, the authors discovered that they made errors in calculating the estimated mean daily case growth rates at 7, 14, and 21 days; which caused them to make errors in calculating the doubling times at 7, 14, and 21 days; which caused them to make errors in calculating the number of expected cases at 7, 14, and 21 days; which caused them to make errors in calculating the difference in expected cases at 7, 14, and 21 days.

In the corrected manuscript, the authors rely on the estimates from the mixed effects linear regression models to estimate mean daily case growth rates at day 4 (one incubation period), day 7, day 14, and day 21, under the assumptions of social distancing measures vs. no social distancing measures. Under the assumption of no social distancing measures, the correct estimated mean daily case growth rates are as follows: day 4 (one incubation period), 30.8%; day 7, 30.3%; day 14, 29.1%; and day 21, 27.9%. The corresponding numbers of expected cases are as follows: day 4 (one incubation period), 12,194; day 7, 27,111; day 14, 166,927; and day 21, 962,256. Under the assumption of social distancing measures, the correct estimated mean daily case growth rates are as follows: day 4 (one incubation period), 31.5%; day 7, 28.2%; day 14, 20.7%, and day 21, 13.1%. The corresponding numbers of expected cases are as follows: day 4 (one incubation period), 12,253; day 7, 26,504; day 14, 118,712; and day 21, 341,509.

In the corrected manuscript, the doubling times are now calculated using the formula ln(2)/ln(1+r/100), where r denotes the mean daily case growth rate. Using the appropriate formula, the estimated doubling times under the assumption of social distancing measures are as follows: day 0, 2.53; day 4 (one incubation period), 2.58; day 7, 2.79; day 14, 3.69; and day 21, 5.65.

In the first paragraph of the Statistical Analysis, we have added text to the paragraph beginning with "We fitted mixed effects linear regression models…", as follows: "We fitted mixed effects linear regression models, specifying the log difference in daily cases as the outcome of interest and including a random effect for state, to allow for within-state correlation of cases over time. Explanatory variables included time in days, implementation period, and a time-by-implementation-period product term. We relied on the estimates from the mixed effects linear regression models to estimate mean daily case growth rates at day 4 (one incubation period), day 7, day 14, and day 21, under the assumptions of social distancing measures vs. no social distancing measures. Doubling times based on these estimated mean daily case growth rates were calculated using the formula ln(2)/ln(1+r/100). This analysis was not conducted as part of a preplanned/registered study protocol…" The remainder of the paragraph is unchanged.

In the final sentence of the second paragraph of Results, the text incorrectly reads: "This estimate corresponds to a mean daily case growth rate that had declined to 26.5% (doubling of total cases every 3.8 days) by day 7 after enactment of the first statewide social distancing measures, to 19.6% (doubling time of 5.1 days) by day 14, and to 12.7% (doubling time of 7.9 days) by day 21."

The corrected text should read: "This estimate corresponds to a mean daily case growth rate that had declined to 28.2% (doubling of total cases every 2.8 days) by day 7 after enactment of the first statewide social distancing measures, to 20.7% (doubling time of 3.7 days) by day 14, and to 13.1% (doubling time of 5.7 days) by day 21."

Beginning with the third sentence of the first paragraph of Discussion, the text incorrectly reads: "Assuming a cumulative epidemic size of 4,125 reported cases (equivalent to the cumulative number of cases in the US at the time of implementation in each state), the reduction in growth rate we estimated corresponds to a difference between 26,281 reported cases with no social distancing versus 24,625 reported cases with social distancing, at 7 days after implementation; a difference between 158,518 reported cases with no social distancing versus 102,223 reported cases with social distancing, at 14 days after implementation; and a difference between 904,773 reported cases with no social distancing versus 283,161 reported cases with social distancing, at 21 days after implementation."

The corrected text should read: "Assuming a cumulative epidemic size of 4,125 reported cases (equivalent to the cumulative number of cases in the US at the time of implementation in each state), the reduction in growth rate we estimated corresponds to a difference between 27,111 reported cases with no social distancing versus 26,504 reported cases with social distancing, at 7 days after implementation; a difference between 166,927 reported cases with no social distancing versus 118,712 reported cases with social distancing, at 14 days after implementation; and a difference between 962,256 reported cases with no social distancing versus 341,509 reported cases with social distancing, at 21 days after implementation."

In the penultimate sentence of the first paragraph of Discussion, the text incorrectly reads: "Stated differently, our model implies that social distancing reduced the total number of reported COVID-19 cases by approximately 1,600 cases at 7 days after implementation, by approximately 56,000 reported cases at 14 days after implementation, and by approximately 621,000 reported cases at 21 days after implementation."

The corrected text should read: "Stated differently, our model implies that social distancing reduced the total number of reported COVID-19 cases by approximately 600 cases at 7 days after implementation, by approximately 48,000 reported cases at 14 days after implementation, and by approximately 621,000 reported cases at 21 days after implementation."

In the Author Summary, the third bullet point under "What did the researchers do and find?" the text incorrectly reads: "Our model implies that social distancing reduced the total number of COVID-19 cases by approximately 1,600 reported cases at 7 days after implementation, by approximately 55,000 reported cases at 14 days after implementation, and by approximately 600,000 reported cases at 21 days after implementation."

The corrected text should read: "Our model implies that social distancing reduced the total number of COVID-19 cases by approximately 600 reported cases at 7 days after implementation, by approximately 48,000 reported cases at 14 days after implementation, and by approximately 621,000 reported cases at 21 days after implementation."

The authors have confirmed that the findings reported in the Abstract and elsewhere in the article text and tables are correctly reported. The authors regret these errors.
