# Supplementary material for: Social distancing to slow the US COVID-19 epidemic: Longitudinal pretest–posttest comparison group study
Source: PLoS Med. 2020 Aug 11;17(8):e1003244. doi: 10.1371/journal.pmed.1003244 (PMC7418951; doi:10.1371/journal.pmed.1003244)
Supplement: S1 Text — (DOCX) [file pmed.1003244.s002.docx]

**S1 Text**

Siedner MJ, Harling G, Reynolds Z, Gilbert RF, Haneuse S, Venkataramani AS, Tsai AC. Social distancing to slow the U.S. COVID-19 epidemic: longitudinal pretest-posttest comparison group study. PLoS Medicine, in press.

**Supplementary Material.** Materials and Methods

**S1 Text Table A.** Dates of implementation of statewide social distancing measures, by type of measure and by state

**S1 Text Table B.** Sensitivity analyses for the effect of implementation of any statewide social distancing measure on daily epidemic growth rate

**S1 Text Table C.** Sensitivity analyses for the effect of implementation of statewide restriction on internal movement on daily epidemic growth rate

**S1 Text Table D.** Sensitivity analyses for the effect of implementation of any statewide social distancing measure on daily epidemic growth rate, with assumed incubation periods of varying duration

**S1 Text Fig A.** Timeline of implementation of first statewide social distancing measures and statewide restrictions on internal movement

**S1 Text Fig B.** Changes in mean daily case growth rate before versus after implementation of the first statewide social distancing measures, in states that implemented such measures versus daily case growth in states that did not implement such measures

**S1 Text Fig C.** Changes in mean daily COVID-19-attributed deaths before versus after implementation of the first statewide social distancing measures, in states that implemented such measures versus daily COVID-19-attributed deaths in states that did not implement such measures

**Supplementary Material.** Materials and Methods

*Identification of statewide social distancing measures*

Our strategy to identify all statewide social distancing measures involved the triangulation of data from state government and third-party sources. First, during March 25-May 1, 2020 we searched the web sites for all 50 U.S. state and commonwealth governments and the District of Columbia to identify all statewide social distancing measures implemented from January 21-May 1, 2020 to address the spread of coronavirus infection, including executive orders; legislation; state of emergency or public health emergency declarations; major disaster declarations; restrictions on travel or internal movement; limitations on mass gatherings; and closures of schools or workplaces. Our searches included the web sites for the respective state governments, state departments of education, state departments of public health, and any state-sponsored COVID-specific web sites.

To ensure the completeness of our search, we then cross-referenced our findings against several third-party sources:

- Al Jazeera Media Network. Lockdowns, closures: How is each U.S. state handling coronavirus? *Al Jazeera*. Available at: https://www.aljazeera.com/news/2020/03/emergencies-closures-states-handling-coronavirus-200317213356419.html. Last accessed April 8, 2020.
- The Council of State Governments. COVID-19 resources for state leaders. Available at: https://web.csg.org/covid19/executive-orders/. Last accessed April 8, 2020.
- Gershman D. A guide to state coronavirus lockdowns. *Wall Street Journal*. Available at: https://www.wsj.com/articles/a-state-by-state-guide-to-coronavirus-lockdowns-11584749351. Last accessed April 8, 2020.
- Mervosh S, Lu D, Swales V. See which states and cities have told residents to stay at home. *New York Times*. Available at: https://www.nytimes.com/interactive/2020/us/coronavirus-stay-at-home-order.html. Last accessed April 30, 2020.
- MultiState. COVID-19 policy tracker. Available at: https://www.multistate.us/pages/covid-19-policy-tracker. Last accessed March 31, 2020.

In the event we identified any discrepancies, official state government sources were assumed to have precedence.

To be included in our analysis, social distancing measures had to meet the following criteria:

1. The social distancing measure had to have been announced and implemented by May 1, 2020.
2. The social distancing measure had to be binding. For example, on March 15, the Governor of Iowa issued a statement recommending that all public schools close for four weeks beginning March 16. On March 17, the Governor then issued a State of Public Health Disaster Emergency that included, among other provisions, mandatory closures of bars, eateries, and other facilities. Thus, in our dataset the state of Iowa was coded as implementing its first statewide social distancing measure on March 17, and the recommendation on school closings was not included.
3. The social distancing measure had to be binding statewide. As another example, on March 13, the Jefferson County (Alabama) Board of Education issued a statement closing all public schools beginning March 16. On the same day, the Governor of Alabama issued a proclamation that public schools would be closed beginning March 18. Thus, in our dataset the state of Alabama was coded as implementing its first statewide social distancing measure on March 18.

We categorized social distancing measures using a previously published typology [1]: closures of schools, closures of workplaces, cancellations of public events, restrictions on internal movement, and closures of state borders. The complete list of dates of implementation of statewide social distancing measures, by type of measure and by state, is shown in **S1 Text Table A**. The timeline of implementation of these measures is shown in **S1 Text Fig A** (figure design adapted from Adolph and colleagues [2]).

*Sensitivity analyses*

In the analyses described in the main text of the manuscript, we examined the effects of two types of statewide social distancing measures. First, we examined the effect of implementing the first statewide social distancing measure (of any kind). Second, we examined the effect of implementing a statewide restriction on internal movement, often referred to as a shelter-in-place order or colloquially referred to as a “lockdown.”

To assess the robustness of our findings, we conducted several sensitivity analyses. First, to adjust for potential confounding by population density, we adjusted our estimates by state-level population density [3,4]. To account for weekly periodicity that could also coincide with implementation of social distancing measures, we also adjusted for day of the week [5,6]. When we adjusted for these covariates, in the analysis estimating the effect of the first statewide social distancing measure of any kind (second row, **S1 Text Table B**) the magnitude of the estimated regression coefficient was reduced slightly but remained statistically significant, implying a reduction in the daily case growth rate of 0.8% per day (b=-0.008; 95% CI, -0.014 to -0.003, *P*<0.001). The estimated effect of statewide restrictions on internal movement, after adjustment for covariates, remained virtually identical to the unadjusted estimate (second row, **S1 Text Table C**).

Second, to assess the extent to which early vs. late implementation of social distancing measures modified the effects of these actions [7], we stratified our estimates by the size of the epidemic in the state. In the analysis estimating the effect of the first statewide social distancing measure of any kind, epidemic size was categorized into <100 versus ≥100 cases at the time the first statewide social distancing measure was implemented. As can be observed in the third and fourth rows of **S1 Text Table B**, the stratified estimates suggested that the reduction in growth rate was more pronounced in states with more than 100 cases at the time of the first lockdown measure. In the analysis estimating the effect of statewide restrictions on internal movement, which were implemented a median of 11 days after the first social distancing measure (when states were farther along in the course of their respective epidemics), epidemic size was categorized into <500 versus ≥500 cases at the time the first statewide restriction on internal movement was implemented. As can be observed in the third and fourth rows of **S1 Text Table C**, neither of the stratified estimates differed meaningfully from the primary model.

Third, although implementation of social distancing measures is most likely to show population-level effectiveness in reducing coronavirus transmission beginning at the lower bound of its estimated incubation period [8,9], we refitted the regression models specifying a range of different incubation periods. As can be observed in **S1 Text Table D**, lengthening the incubation period generally attenuated both the magnitude and statistical significance of the estimated regression coefficient on the primary variable of interest. There was also a strong suggestion of a signal extending back to day 0, consistent with the possibility that individual behavior change could have begun to occur even prior to the introduction of statewide social distancing measures, either in response to local (e.g., county-level) social distancing measures that were enacted prior the first statewide measures, or in response to generalized fears about the epidemic resulting in spontaneous behavior change even in the absence of any state or local policies [10].

Fourth, to assess the extent to which our estimates may have been driven by model specification, we replaced the longitudinal pretest-posttest comparison group study design with a multivariable regression model in which the primary explanatory variables of interest were a series of binary indicators denoting each day before versus after implementation of the first statewide social distancing measures (often described in the econometrics literature as an event study specification [11-13]). This approach compares daily case growth before versus after implementation of the first statewide social distancing measures in states that implemented such measures against daily case growth in states that did not implement such measures. Our regression model included state fixed effects, to adjust for potential confounding from time-invariant state-level factors or baseline differences in population socioeconomic or health characteristics; and linear and quadratic terms for time (days) to adjust for nationwide secular trends in the outcomes. We computed 95% confidence intervals adjusted for clustering within states, the geographical level at which exposure occurred [14]. As can be observed in **S1 Text Fig B**, mean daily case growth was negative by day 4, and the estimates were statistically significant by day 8, consistent with the primary analysis. The event study analysis for change in daily COVID-19-attributed deaths also produced estimates qualitatively similar to the primary analysis, although with slightly larger confidence intervals given the smaller number of events (**S1 Text Fig C**).

*Prespecified analysis plan*

No prospective protocol was published or registered for this observational study. However, we followed a clear analysis plan, as described in the methods section, with minor changes as detailed below.

- The inclusion/exclusion criteria for the study were established at the outset. The initial analysis, described in the manuscript we deposited with the *medRxiv* preprint server on April 8 [15], included data on statewide social distancing measures implemented between January 21 and March 30, 2020, and COVID-19 cases through March 31. Prior to submission of the current manuscript, we updated the dataset and extended the study period to include social distancing measures implemented up to April 8 and COVID-19 cases and deaths up to April 8. In response to editorial feedback, in the first revision of this manuscript we have further updated the dataset and extended the study period to include social distancing measures implemented up to May 1 and COVID-19 cases and deaths up to May 26.
- The outcome of COVID-19 case growth was prespecified at the outset. After deposition of the manuscript with medRxiv preprint server on April 8, given the rapidly expanding epidemic, we expanded the analysis to include growth in COVID-19-attributed deaths as a secondary outcome. This analysis was described in the initially submitted manuscript.
- The statistical analyses were determined at the outset and were not changed. After submission, and in response to reviewer feedback, we added sensitivity analyses to explore the robustness of our findings to alternative specifications.
- The subgroup analyses (namely, stratification by epidemic size at the time of implementation of the first statewide social distancing measures and statewide restrictions on internal movement) were pre-specified and theoretically motivated.

**S1 Text Table A.** Dates of implementation of statewide social distancing measures, by type of measure and by state

| **State** | **Date** | **A** | **B** | **C** | **D** | **E** | **URL** |
| --- | --- | --- | --- | --- | --- | --- | --- |
| Alabama | 3/18/20 | 🗸 |  |  |  |  | - https://governor.alabama.gov/newsroom/2020/03/supplemental-state-of-emergency-coronavirus-covid-19/ - https://www.alsde.edu/COVID19%20Updates/4thSupplementalStateofEmergencyCOVID-19.pdf |
| Alabama | 3/19/20 | 🗸 |  | 🗸 |  |  | - https://governor.alabama.gov/assets/2020/03/Alabama-State-Health-Officer-Statewide-Social-Distancing-Order-3.19.20.pdf - https://governor.alabama.gov/assets/2020/03/Amended-Statewide-Social-Distancing-SHO-Order-3.27.2020-FINAL.pdf |
| Alabama | 3/28/20 |  | 🗸 |  |  |  | - <https://governor.alabama.gov/assets/2020/03/Amended-Statewide-Social-Distancing-SHO-Order-3.27.2020-FINAL.pdf> |
| Alabama | 4/4/20 |  |  |  | 🗸 |  | - <https://governor.alabama.gov/assets/2020/04/Final-Statewide-Order-4.3.2020.pdf> |
| Alaska | 3/14/20 |  |  | 🗸 |  |  | - <https://gov.alaska.gov/wp-content/uploads/sites/2/03132020-COVID-19-Health-Mandate-001.pdf> |
| Alaska | 3/16/20 | 🗸 |  |  |  |  | - https://gov.alaska.gov/home/covid19-healthmandates/ - https://gov.alaska.gov/wp-content/uploads/sites/2/03132020-COVID-19-Health-Mandate-001.pdf |
| Alaska | 3/17/20 |  |  | 🗸 |  | 🗸 | - <https://gov.alaska.gov/home/covid19-healthmandates/> |
| Alaska | 3/18/20 |  |  | 🗸 |  |  | - <https://gov.alaska.gov/home/covid19-healthmandates/> |
| Alaska | 3/20/20 | 🗸 |  |  |  |  | - <https://gov.alaska.gov/home/covid19-healthmandates/> |
| Alaska | 3/24/20 |  | 🗸 | 🗸 |  |  | - <https://gov.alaska.gov/home/covid19-healthmandates/> |
| Alaska | 3/25/20 |  |  |  |  | 🗸 | - <https://gov.alaska.gov/home/covid19-healthmandates/> |
| Alaska | 3/28/20 |  |  |  | 🗸 |  | - <https://gov.alaska.gov/wp-content/uploads/sites/2/03272020-SOA-COVID-19-Health-Mandate-011.pdf> |
| Arizona | 3/16/20 | 🗸 |  |  |  |  | - https://azgovernor.gov/governor/blog/2020/03/open-letter-arizona-families-educators-school-leaders-and-education-community - https://azgovernor.gov/governor/news/2020/03/governor-ducey-superintendent-hoffman-announce-extension-school-closures |
| Arizona | 3/20/20 |  |  | 🗸 |  |  | - <https://azgovernor.gov/sites/default/files/eo_2020-09_3.pdf> |
| Arizona | 3/31/20 |  | 🗸 |  | 🗸 |  | - https://www.azdhs.gov/documents/preparedness/epidemiology-disease-control/infectious-disease-epidemiology/novel-coronavirus/eo-stay-home-stay-healthy-stay-connected.pdf - https://azgovernor.gov/sites/default/files/eo_2020-33_0.pdf |
| Arizona | 4/9/20 |  |  |  |  | 🗸 | - <https://azgovernor.gov/sites/default/files/eo-2020-24.pdf> |
| Arkansas | 3/19/20 | 🗸 | 🗸 | 🗸 |  |  | - https://www.healthy.arkansas.gov/images/uploads/pdf/Directive_03.19.2020_final.pdf - https://governor.arkansas.gov/images/uploads/executiveOrders/EO_20-13._.pdf - https://governor.arkansas.gov/news-media/press-releases/governor-hutchinson-announces-all-schools-to-remain-closed-for-remainder-of |
| Arkansas | 3/25/20 |  |  | 🗸 |  |  | - <https://www.healthy.arkansas.gov/images/uploads/pdf/Barber_Body_Art_Cosmetology_Massage_Therapy_Medical_Spas_Schools_FINAL.pdf> |
| Arkansas | 3/27/20 |  |  | 🗸 |  |  | - <https://governor.arkansas.gov/images/uploads/executiveOrders/EO_20-10._.pdf> |
| California | 3/11/20 |  |  | 🗸 |  |  | - <https://www.gov.ca.gov/2020/03/11/california-public-health-experts-mass-gatherings-should-be-postponed-or-canceled-statewide-to-slow-the-spread-of-covid-19/> |
| California | 3/17/20 |  |  | 🗸 |  |  | - <https://www.cdph.ca.gov/Programs/CID/DCDC/CDPH%20Document%20Library/COVID-19/Coronavirus%20Disease%202019%20and%20Food%20Beverage%20Other%20Services%20-%20AOL.pdf> |
| California | 3/19/20 | 🗸 | 🗸 |  | 🗸 |  | - <https://www.gov.ca.gov/wp-content/uploads/2020/03/3.19.20-attested-EO-N-33-20-COVID-19-HEALTH-ORDER.pdf> |
| Colorado | 3/17/20 |  |  | 🗸 |  |  | - https://www.colorado.gov/pacific/sites/default/files/atoms/files/Bars%20Restaurants%20PH%20order.pdf - https://www.colorado.gov/pacific/sites/default/files/atoms/files/Amended%20Bars%20Restaurants%20PH%20order.20-22%20%281%29.pdf |
| Colorado | 3/19/20 |  |  | 🗸 |  |  | - https://covid19.colorado.gov/public-health-executive-orders-explained - https://drive.google.com/file/d/14X5zbYPY7LJ8zzzSv_GcApJauFmsc5Ju/view |
| Colorado | 3/23/20 | 🗸 |  | 🗸 |  |  | - https://drive.google.com/file/d/1ecMEQj3F3qeEl3qNMtLkAlk3ya3FbVH3/view - https://drive.google.com/file/d/1KNqVxX8m2Y_cInIMSqIZeMoxIlAFtNo0/view - https://www.colorado.gov/governor/sites/default/files/inline-files/D%202020%20021%20P-12%20Extension_0.pdf - https://ewscripps.brightspotcdn.com/7f/e5/67a8f6db4d49a86021a9dc4f0bc1/d-2020-041-p-12-closure-extension-end-of-year.pdf |
| Colorado | 3/26/20 |  | 🗸 |  | 🗸 |  | - https://drive.google.com/file/d/1O1EDCY6-A6QBKxzDImCSF8bBBdOOI3Km/view - https://www.colorado.gov/governor/sites/default/files/inline-files/D%202020%20024%20Amending%20and%20Extending%20Executive%20Order%20D%202020%20017%20Stay%20at%20Home%20Order_0.pdf |
| Connecticut | 3/12/20 |  |  | 🗸 |  |  | - https://portal.ct.gov/-/media/Office-of-the-Governor/Executive-Orders/Lamont-Executive-Orders/Executive-Order-No-7.pdf |
| Connecticut | 3/16/20 |  |  | 🗸 |  |  | - https://portal.ct.gov/-/media/Office-of-the-Governor/Executive-Orders/Lamont-Executive-Orders/Executive-Order-No-7D.pdf - https://portal.ct.gov/-/media/Office-of-the-Governor/Executive-Orders/Lamont-Executive-Orders/Executive-Order-No-7X.pdf |
| Connecticut | 3/17/20 | 🗸 |  |  |  |  | - https://portal.ct.gov/-/media/Office-of-the-Governor/Executive-Orders/Lamont-Executive-Orders/Executive-Order-No-7C.pdf - https://portal.ct.gov/-/media/Office-of-the-Governor/Executive-Orders/Lamont-Executive-Orders/Executive-Order-No-7X.pdf |
| Connecticut | 3/19/20 |  |  | 🗸 |  |  | - https://portal.ct.gov/-/media/Office-of-the-Governor/Executive-Orders/Lamont-Executive-Orders/Executive-Order-No-7F.pdf - https://portal.ct.gov/-/media/Office-of-the-Governor/Executive-Orders/Lamont-Executive-Orders/Executive-Order-No-7X.pdf |
| Connecticut | 3/23/20 |  | 🗸 |  | 🗸 |  | - <https://portal.ct.gov/Office-of-the-Governor/News/Press-Releases/2020/03-2020/Governor-Lamont-Releases-Guidance-to-Businesses-on-Order-Asking-Connecticut-to-Stay-Safe-Stay-Home> |
| Connecticut | 3/26/20 |  |  | 🗸 |  |  | - https://portal.ct.gov/-/media/Office-of-the-Governor/Executive-Orders/Lamont-Executive-Orders/Executive-Order-No-7N.pdf - https://portal.ct.gov/-/media/Office-of-the-Governor/Executive-Orders/Lamont-Executive-Orders/Executive-Order-No-7X.pdf |
| Delaware | 3/16/20 | 🗸 |  | 🗸 |  |  | - https://governor.delaware.gov/wp-content/uploads/sites/24/2020/03/School-Letter-Governor-Carney-03132020.pdf - https://governor.delaware.gov/wp-content/uploads/sites/24/2020/03/Second-Modification-to-the-State-of-Emergency.pdf - https://governor.delaware.gov/health-soe/tenth-state-of-emergency/ |
| Delaware | 3/24/20 |  | 🗸 |  | 🗸 |  | - https://governor.delaware.gov/wp-content/uploads/sites/24/2020/03/Fifth-Modification-to-State-of-Emergency-03222020.pdf - https://governor.delaware.gov/health-soe/tenth-state-of-emergency/ |
| Delaware | 3/30/20 |  |  |  |  | 🗸 | - <https://governor.delaware.gov/health-soe/seventh-state-of-emergency/> |
| Delaware | 4/2/20 |  |  | 🗸 |  |  | - <https://governor.delaware.gov/health-soe/ninth-state-of-emergency/> |
| District of Columbia | 3/13/20 |  |  | 🗸 |  |  | - <https://coronavirus.dc.gov/sites/default/files/dc/sites/coronavirus/release_content/attachments/DOH_Rulemaking_Mass-Gatherings.pdf> |
| District of Columbia | 3/16/20 | 🗸 |  | 🗸 |  |  | - https://mayor.dc.gov/sites/default/files/dc/sites/mayormb/publication/attachments/MO-Prohibition-on-Mass-Gatherings-During-Public-Health-Emergency.pdf - https://dcps.dc.gov/sites/default/files/dc/sites/dcps/page_content/attachments/DCPS_SchoolClosure_031320_Final_English.pdf - https://dcps.dc.gov/sites/default/files/dc/sites/dcps/page_content/attachments/04172020_Calendar_Update_Final_English.pdf |
| District of Columbia | 3/25/20 |  | 🗸 |  |  |  | - https://coronavirus.dc.gov/sites/default/files/dc/sites/mayormb/release_content/attachments/Mayor%27s%20Order%202020-053%20Closure%20of%20Non-Essential%20Businesses%20and%20Prohibiti....pdf - https://coronavirus.dc.gov/sites/default/files/dc/sites/coronavirus/publication/attachments/MayorsOrder2020.063.pdf |
| District of Columbia | 4/1/20 |  |  |  | 🗸 |  | - https://mayor.dc.gov/release/mayor-bowser-issues-stay-home-order - https://coronavirus.dc.gov/sites/default/files/dc/sites/coronavirus/publication/attachments/MayorsOrder2020.063.pdf |
| Florida | 3/16/20 | 🗸 |  |  |  |  | - http://www.fldoe.org/core/fileparse.php/19861/urlt/Florida-2020-BreakDates.pdf - http://www.fldoe.org/newsroom/latest-news/florida-department-of-education-announces-additional-guidance-for-the-2019-20-school-year.stml - http://www.fldoe.org/newsroom/latest-news/florida-extends-school-campus-closures-through-may-1.stml - http://www.fldoe.org/newsroom/latest-news/florida-extends-distance-learning-through-remainder-of-academic-year.stml |
| Florida | 3/17/20 |  |  | 🗸 |  |  | - <https://www.flgov.com/wp-content/uploads/2020/03/EO-20-68.pdf> |
| Florida | 3/20/20 |  |  | 🗸 |  |  | - <https://www.flgov.com/wp-content/uploads/orders/2020/EO_20-71.pdf> |
| Florida | 3/24/20 |  |  |  |  | 🗸 | - https://www.flgov.com/wp-content/uploads/2020/03/EO-20-80.pdf - https://www.flgov.com/wp-content/uploads/orders/2020/EO_20-82.pdf - https://www.flgov.com/wp-content/uploads/orders/2020/EO_20-86.pdf - https://www.flgov.com/wp-content/uploads/orders/2020/EO_20-52.pdf - https://www.flgov.com/wp-content/uploads/orders/2020/EO_20-114.pdf |
| Florida | 4/3/20 |  | 🗸 |  | 🗸 |  | - <https://www.flgov.com/wp-content/uploads/orders/2020/EO_20-91-compressed.pdf> |
| Georgia | 3/18/20 | 🗸 |  |  |  |  | - <https://gov.georgia.gov/executive-action/executive-orders/2020-executive-orders> |
| Georgia | 3/24/20 |  |  | 🗸 | 🗸 |  | - https://gov.georgia.gov/document/2020-executive-order/03232001/download - https://gov.georgia.gov/executive-action/executive-orders/2020-executive-orders |
| Georgia | 4/3/20 |  | 🗸 |  | 🗸 |  | - <https://gov.georgia.gov/executive-action/executive-orders/2020-executive-orders> |
| Hawaii | 3/15/20 | 🗸 |  |  |  |  | - http://www.hawaiipublicschools.org/DOE%20Forms/Emergencies/2020-03-15-Spring-Break-Extension-All-Schools-LTP.pdf - http://www.hawaiipublicschools.org/ConnectWithUs/MediaRoom/PressReleases/Pages/School-facilities-closed-to-students-through-April-30.aspx - http://www.hawaiipublicschools.org/ConnectWithUs/MediaRoom/PressReleases/Pages/HIDOE-enrichment-and-distance-learning-to-continue-for-the-remainder-of-the-2019-20-school-year.aspx |
| Hawaii | 3/18/20 |  |  | 🗸 |  |  | - <https://governor.hawaii.gov/newsroom/latest-news/dlnr-news-release-covid-19-leads-to-parks-and-facilities-closures-march-17-2020/> |
| Hawaii | 3/20/20 |  |  | 🗸 |  |  | - <https://governor.hawaii.gov/newsroom/latest-news/proper-use-of-covid-19-tests-imperative-there-is-a-current-shortage-of-hand-sanitizers-and-toilet-paper-in-hawaii-in-part-because-of-the-publics-over-reaction-to-covid-19-the-hawai/> |
| Hawaii | 3/25/20 |  | 🗸 |  | 🗸 |  | - https://governor.hawaii.gov/newsroom/latest-news/office-of-the-governor-news-release-governor-ige-issues-statewide-order-to-stay-at-home-work-from-home-to-fight-covid-19/ - https://hawaiicovid19.com/wp-content/uploads/2020/03/2003162-ATG_Third-Supplementary-Proclamation-for-COVID-19-signed-12.pdf - https://governor.hawaii.gov/wp-content/uploads/2020/04/2004144-ATG_Sixth-Supplementary-Proclamation-for-COVID-19-distribution-signed.pdf |
| Hawaii | 3/26/20 |  |  |  |  | 🗸 | - <https://governor.hawaii.gov/wp-content/uploads/2020/03/2003152-ATG_Second-Supplementary-Proclamation-for-COVID-19-signed.pdf> |
| Idaho | 3/25/20 | 🗸 | 🗸 |  | 🗸 |  | - https://coronavirus.idaho.gov/wp-content/uploads/sites/127/2020/04/amended-statewide-stay-home-order_041520.pdf - https://www.sde.idaho.gov/ |
| Illinois | 3/13/20 |  |  | 🗸 |  |  | - <https://www2.illinois.gov/Documents/ExecOrders/2020/ExecutiveOrder-2020-04.pdf> |
| Illinois | 3/16/20 |  |  | 🗸 |  |  | - https://www2.illinois.gov/Documents/ExecOrders/2020/ExecutiveOrder-2020-07.pdf - https://www2.illinois.gov/Pages/Executive-Orders/ExecutiveOrder2020-18.aspx |
| Illinois | 3/17/20 | 🗸 |  |  |  |  | - https://www2.illinois.gov/Documents/ExecOrders/2020/ExecutiveOrder-2020-05.pdf - https://www2.illinois.gov/Pages/Executive-Orders/ExecutiveOrder2020-18.aspx - https://www2.illinois.gov/Pages/news-item.aspx?ReleaseID=21418 |
| Illinois | 3/21/20 |  | 🗸 |  | 🗸 |  | - https://www2.illinois.gov/Documents/ExecOrders/2020/ExecutiveOrder-2020-10.pdf - https://www2.illinois.gov/Pages/Executive-Orders/ExecutiveOrder2020-18.aspx - https://www2.illinois.gov/Pages/news-item.aspx?ReleaseID=21459 |
| Indiana | 3/16/20 |  |  | 🗸 |  |  | - https://www.in.gov/gov/files/ExecutiveOrder20-04FurtherOrdersforPublicHealthEmergency.pdf - https://www.in.gov/gov/files/Executive_Order_20-10_Enforcement_of%20In_Person%20Dining_Prohibition.pdf - https://www.in.gov/gov/files/Executive%20Order%2020-14%20Continuation%20of%20Restaurant%20Alcohol%20EOs.pdf - https://www.in.gov/gov/files/Executive%20Order%2020-18%20Cont%20Stay%20at%20Home%20Restaurants%20Govt%20Ops.pdf - https://www.in.gov/gov/files/Executive%20Order%2020-22%20Extension%20of%20Stay%20at%20Home.pdf - https://www.in.gov/gov/files/Executive%20Order%2020-26%20Roadmap%20to%20Reopen%20Indiana.pdf |
| Indiana | 3/19/20 | 🗸 |  |  |  |  | - https://www.in.gov/gov/files/EO_20-05.pdf - https://www.in.gov/gov/files/Executive%20Order%2020-16%20Education.pdf |
| Indiana | 3/24/20 |  | 🗸 |  | 🗸 |  | - https://www.in.gov/gov/files/Executive_Order_20-08_Stay_at_Home.pdf - https://www.in.gov/gov/files/Executive%20Order%2020-18%20Cont%20Stay%20at%20Home%20Restaurants%20Govt%20Ops.pdf - https://www.in.gov/gov/files/Executive%20Order%2020-22%20Extension%20of%20Stay%20at%20Home.pdf - https://www.in.gov/gov/files/Executive%20Order%2020-26%20Roadmap%20to%20Reopen%20Indiana.pdf |
| Iowa | 3/17/20 |  |  | 🗸 |  |  | - https://governor.iowa.gov/sites/default/files/documents/Public%20Health%20Proclamation%20-%202020.03.17.pdf - https://governor.iowa.gov/sites/default/files/documents/Public%20Health%20Disaster%20Proclamation%20-%202020.04.02.pdf |
| Iowa | 3/22/20 |  |  | 🗸 |  |  | - https://governor.iowa.gov/sites/default/files/documents/Public%20Health%20Proclamation%20-%202020.03.22.pdf - https://governor.iowa.gov/sites/default/files/documents/Public%20Health%20Proclamation%20-%202020.04.06.pdf |
| Iowa | 3/26/20 |  |  | 🗸 |  |  | - https://governor.iowa.gov/sites/default/files/documents/Public%20Health%20Proclamation%20-%202020.03.26.pdf - https://governor.iowa.gov/sites/default/files/documents/Public%20Health%20Proclamation%20-%202020.04.16%20-%20Region%206.pdf |
| Iowa | 4/2/20 | 🗸 |  |  |  |  | - https://governor.iowa.gov/sites/default/files/documents/Public%20Health%20Disaster%20Proclamation%20-%202020.04.02.pdf - https://governor.iowa.gov/press-release/iowa-schools-to-extend-closures-through-end-of-school-year-schools-will-continue - https://governor.iowa.gov/sites/default/files/documents/Public%20Health%20Proclamation%20-%202020.04.27%20-%20Pt%201.pdf |
| Kansas | 3/17/20 | 🗸 |  | 🗸 |  |  | - https://governor.kansas.gov/wp-content/uploads/2020/03/20-04-Executed.pdf - https://governor.kansas.gov/wp-content/uploads/2020/03/EO-20-07-Executed.pdf - https://governor.kansas.gov/wp-content/uploads/2020/04/EO-20-28-Reissuing-and-Extending-Certain-EOs.pdf |
| Kansas | 3/19/20 |  |  |  |  | 🗸 | - https://khap2.kdhe.state.ks.us/NewsRelease/PDFs/3-19-20%203-19-20%20REVISED%20Mandate%20for%20quarantine%20and%20isolation%20of%20travelers,%20close%20contacts,%20those%20being%20tested.pdf - https://khap2.kdhe.state.ks.us/NewsRelease/PDFs/3-23-2020%20Updates.pdf |
| Kansas | 3/25/20 |  |  | 🗸 |  |  | - https://governor.kansas.gov/wp-content/uploads/2020/03/EO-20-14-Executed.pdf - https://governor.kansas.gov/wp-content/uploads/2020/04/EO-20-25-Executed.pdf - https://governor.kansas.gov/wp-content/uploads/2020/04/EO-20-29-Implementing-Phase-One-of-Ad-Astra-Plan.pdf |
| Kansas | 3/30/20 |  | 🗸 |  | 🗸 |  | - https://governor.kansas.gov/wp-content/uploads/2020/03/EO20-16.pdf - https://governor.kansas.gov/wp-content/uploads/2020/04/EO-20-24-Executed.pdf |
| Kentucky | 3/18/20 |  |  | 🗸 |  |  | - <https://governor.ky.gov/attachments/20200317_Order_Public-Facing-Businesses.pdf> |
| Kentucky | 3/19/20 |  |  | 🗸 |  |  | - <https://governor.ky.gov/attachments/20200319_Order_Mass-Gatherings.pdf> |
| Kentucky | 3/23/20 |  | 🗸 |  |  |  | - <https://governor.ky.gov/attachments/20200322_Executive-Order_2020-246_Retail.pdf> |
| Kentucky | 3/30/20 |  |  |  |  | 🗸 | - <https://governor.ky.gov/attachments/20200330_Executive-Order_2020-258_Out-of-State-Travel.pdf> |
| Louisiana | 3/13/20 | 🗸 |  |  |  |  | - https://www.louisianabelieves.com/docs/default-source/covid-19-resources/27-jbe-2020-covid-19.pdf?sfvrsn=4dfd9b1f_4 4/30/2020 - https://gov.louisiana.gov/assets/Proclamations/2020/52-JBE-2020-Stay-at-Home-Order.pdf |
| Louisiana | 3/17/20 |  |  | 🗸 |  |  | - https://gov.louisiana.gov/assets/Proclamations/2020/Proc-No-30-updTED.pdf - https://gov.louisiana.gov/assets/Proclamations/2020/41-JBE-2020-Stay-At-Home-Extended.pdf |
| Louisiana | 3/23/20 |  | 🗸 |  | 🗸 |  | - https://gov.louisiana.gov/assets/Proclamations/2020/33-JBE-2020-Public-Health-Emergency.pdf - https://gov.louisiana.gov/assets/Proclamations/2020/41-JBE-2020-Stay-At-Home-Extended.pdf - https://gov.louisiana.gov/assets/Proclamations/2020/52-JBE-2020-Stay-at-Home-Order.pdf |
| Maine | 3/18/20 |  |  | 🗸 |  |  | - https://www.maine.gov/governor/mills/sites/maine.gov.governor.mills/files/inline-files/EO%2014%20An%20Order%20to%20Protect%20Public%20Health.pdf - https://www.maine.gov/governor/mills/sites/maine.gov.governor.mills/files/inline-files/An%20Order%20to%20Stay%20Safer%20at%20Home.pdf |
| Maine | 3/25/20 |  | 🗸 |  |  |  | - https://www.maine.gov/governor/mills/sites/maine.gov.governor.mills/files/inline-files/An%20Order%20Regarding%20Essential%20Businesses%20and%20Operations%20_0.pdf - https://www.maine.gov/governor/mills/sites/maine.gov.governor.mills/files/inline-files/An%20Order%20Regarding%20Further%20Restrictions%20on%20Public%20Contact%20and%20Movement%2C%20Schools%2C%20Vehicle%20Travel%20and%20Retail%20Business%20Operations.pdf - https://www.maine.gov/governor/mills/sites/maine.gov.governor.mills/files/inline-files/An%20Order%20to%20Stay%20Safer%20at%20Home.pdf |
| Maine | 4/2/20 | 🗸 |  |  | 🗸 |  | - https://www.maine.gov/governor/mills/sites/maine.gov.governor.mills/files/inline-files/An%20Order%20Regarding%20Further%20Restrictions%20on%20Public%20Contact%20and%20Movement%2C%20Schools%2C%20Vehicle%20Travel%20and%20Retail%20Business%20Operations.pdf - https://www.maine.gov/governor/mills/sites/maine.gov.governor.mills/files/inline-files/An%20Order%20to%20Stay%20Safer%20at%20Home.pdf |
| Maine | 4/3/20 |  |  |  |  | 🗸 | - https://www.maine.gov/governor/mills/sites/maine.gov.governor.mills/files/inline-files/EO%2034.pdf - https://www.maine.gov/governor/mills/sites/maine.gov.governor.mills/files/inline-files/An%20Order%20to%20Stay%20Safer%20at%20Home.pdf |
| Maryland | 3/12/20 |  |  | 🗸 |  |  | - <https://governor.maryland.gov/wp-content/uploads/2020/03/Prohibiting-Large-Gatherings.pdf> |
| Maryland | 3/16/20 | 🗸 |  | 🗸 |  |  | - https://governor.maryland.gov/wp-content/uploads/2020/03/Prohibiting-Large-Gatherings.pdf - https://governor.maryland.gov/wp-content/uploads/2020/03/Executive-Order-Amending-Large-Gatherings.pdf - http://marylandpublicschools.org/Pages/default.aspx |
| Maryland | 3/23/20 |  | 🗸 |  |  |  | - <https://governor.maryland.gov/wp-content/uploads/2020/03/Gatherings-THIRD-AMENDED-3.23.20.pdf> |
| Maryland | 3/30/20 |  | 🗸 |  | 🗸 |  | - <https://governor.maryland.gov/wp-content/uploads/2020/03/Gatherings-FOURTH-AMENDED-3.30.20.pdf> |
| Massachusetts | 3/13/20 |  |  | 🗸 |  |  | - <https://www.mass.gov/doc/order-prohibiting-gatherings-of-more-than-250-people/download> |
| Massachusetts | 3/16/20 | 🗸 |  |  |  |  | - https://www.mass.gov/doc/march-16-2020-k-12-school-closing-order/download - https://www.mass.gov/doc/march-25-school-closure-extension-order - https://www.mass.gov/doc/april-21-2020-school-closure-extension-order |
| Massachusetts | 3/17/20 |  |  | 🗸 |  |  | - https://www.mass.gov/doc/march-15-2020-large-gatherings-25-and-restaurants-order - https://www.mass.gov/doc/march-31-2020-essential-services-extension-order - https://www.mass.gov/doc/signed-second-extension-of-essential-services-order/download |
| Massachusetts | 3/24/20 |  | 🗸 |  | 🗸 |  | - https://www.mass.gov/doc/march-23-2020-essential-services-and-revised-gatherings-order - https://www.mass.gov/news/governor-charlie-baker-orders-all-non-essential-businesses-to-cease-in-person-operation - https://www.mass.gov/info-details/covid-19-essential-services |
| Massachusetts | 3/27/20 |  |  |  |  | 🗸 | - <https://www.mass.gov/info-details/travel-information-related-to-covid-19> |
| Michigan | 3/16/20 | 🗸 |  |  |  |  | - https://www.michigan.gov/whitmer/0,9309,7-387-90499_90705-521595--,00.html - https://www.michigan.gov/whitmer/0,9309,7-387-90499_90705-524032--,00.html - https://www.michigan.gov/whitmer/0,9309,7-387-90499_90705-527719--,00.html |
| Michigan | 3/22/20 |  |  | 🗸 |  |  | - https://www.michigan.gov/whitmer/0,9309,7-387-90499_90705-522576--,00.html - https://www.michigan.gov/whitmer/0,9309,7-387-90499_90705-525927--,00.html |
| Michigan | 3/24/20 |  | 🗸 |  | 🗸 |  | - https://www.michigan.gov/whitmer/0,9309,7-387-90499_90705-522626--,00.html - https://www.michigan.gov/whitmer/0,9309,7-387-90499_90705-525182--,00.html - https://www.michigan.gov/whitmer/0,9309,7-387-90499_90705-526894--,00.html - https://www.michigan.gov/whitmer/0,9309,7-387-90499_90705-527847--,00.html - https://www.michigan.gov/whitmer/0,9309,7-387-90499_90705-528460--,00.html |
| Minnesota | 3/17/20 |  |  | 🗸 |  |  | - https://mn.gov/governor/assets/2020_03_16_EO_20_04_Bars_Restaurants_tcm1055-423380.pdf - https://www.leg.state.mn.us/archive/execorders/20-33.pdf - https://www.leg.state.mn.us/archive/execorders/20-48.pdf |
| Minnesota | 3/18/20 | 🗸 |  |  |  |  | - https://www.leg.state.mn.us/archive/execorders/20-02.pdf - https://www.leg.state.mn.us/archive/execorders/20-41.pdf |
| Minnesota | 3/27/20 |  | 🗸 |  | 🗸 |  | - http://mn.gov/governor/covid-19/news/index.jsp?id=1055-424820 - https://www.leg.state.mn.us/archive/execorders/20-33.pdf - https://www.leg.state.mn.us/archive/execorders/20-48.pdf |
| Mississippi | 3/19/20 | 🗸 |  |  |  |  | - https://www.sos.ms.gov/Education-Publications/ExecutiveOrders/1460.pdf - https://www.sos.ms.gov/Pages/Governor-Tate-Reeves-Announces-Extended-School-Closures-to-Slow-the-Spread.aspx |
| Mississippi | 3/24/20 |  |  | 🗸 |  |  | - <https://www.sos.ms.gov/Education-Publications/ExecutiveOrders/1463.pdf> |
| Mississippi | 3/31/20 |  | 🗸 |  | 🗸 |  | - https://www.sos.ms.gov/Education-Publications/ExecutiveOrders/1465.pdf - https://www.sos.ms.gov/Education-Publications/ExecutiveOrders/1466.pdf |
| Missouri | 3/17/20 |  |  | 🗸 |  |  | - <https://governor.mo.gov/press-releases/archive/what-you-need-know-missouris-guidelines-combat-spread-covid-19> |
| Missouri | 3/19/20 | 🗸 |  |  |  |  | - https://dese.mo.gov/communications/coronavirus-covid-19-information - https://dese.mo.gov/sites/default/files/Gov-Closes-Schools-April-9.pdf - https://governor.mo.gov/priorities/extension-stay-home-order-covd-19 |
| Missouri | 3/23/20 |  |  | 🗸 |  |  | - <https://governor.mo.gov/press-releases/archive/governor-parson-directs-dhss-director-require-social-distancing-statewide> |
| Missouri | 3/23/20 |  |  | 🗸 |  |  | - https://governor.mo.gov/press-releases/archive/governor-parson-directs-dhss-director-require-social-distancing-statewide - https://governor.mo.gov/priorities/stay-home-order - https://governor.mo.gov/priorities/extension-stay-home-order-covd-19 |
| Missouri | 4/6/20 |  | 🗸 |  | 🗸 |  | - https://governor.mo.gov/priorities/stay-home-order - https://governor.mo.gov/priorities/extension-stay-home-order-covd-19 |
| Montana | 3/16/20 | 🗸 |  |  |  |  | - http://governor.mt.gov/Pressroom/governor-bullock-directs-the-closure-of-public-k-12-schools-for-two-weeks-strongly-recommends-social-distancing-measures-to-slow-the-spread-of-covid-19 - http://governor.mt.gov/Portals/16/Closure%20Extensions%20and%20Social%20Distancing.pdf?ver=2020-03-24-164313-497 |
| Montana | 3/20/20 |  |  | 🗸 |  |  | - <http://governor.mt.gov/Pressroom/governor-bullock-announces-closure-of-dine-in-food-service-and-alcoholic-beverage-businesses-and-other-activities-that-pose-enhanced-risks-to-curtail-spread-of-covid-19> |
| Montana | 3/28/20 |  | 🗸 |  | 🗸 |  | - https://covid19.mt.gov/Portals/223/Documents/Stay%20at%20Home%20Directive.pdf?ver=2020-03-26-173332-177 - http://governor.mt.gov/Pressroom/governor-bullock-extends-directives-issued-to-respond-to-covid-19-pandemic - https://covid19.mt.gov/Portals/223/Documents/04-22-20%20Directive%20and%20Appx%20-%20Reopening%20Phase%20One.pdf?ver=2020-04-22-124954-977 |
| Montana | 3/30/20 |  |  |  |  | 🗸 | - http://governor.mt.gov/Portals/16/Quarantine%20for%20Travelers.pdf?ver=2020-03-30-170637-190 - http://governor.mt.gov/Pressroom/governor-bullock-extends-directives-issued-to-respond-to-covid-19-pandemic - https://covid19.mt.gov/Portals/223/Documents/04-22-20%20Directive%20and%20Appx%20-%20Reopening%20Phase%20One.pdf?ver=2020-04-22-124954-977 |
| Nebraska | 3/16/20 |  |  | 🗸 |  |  | - <https://governor.nebraska.gov/press/gov-ricketts-further-limits-events-gatherings-prevent-covid-19-spread> |
| Nebraska | 4/3/20 | 🗸 |  |  |  |  | - https://governor.nebraska.gov/press/gov-ricketts-announces-15-counties-north-and-central-nebraska-now-included-state%E2%80%99s-directed - https://governor.nebraska.gov/press/gov-ricketts-adds-additional-businesses-state%E2%80%99s-directed-health-measure - https://www.dropbox.com/s/xh4pyr4w56g3tdh/DHM%204.9.2020.pdf |
| Nebraska | 4/10/20 | 🗸 |  | 🗸 |  |  | - https://governor.nebraska.gov/press/gov-ricketts-adds-additional-businesses-state%E2%80%99s-directed-health-measure - https://www.dropbox.com/s/xh4pyr4w56g3tdh/DHM%204.9.2020.pdf |
| Nevada | 3/16/20 | 🗸 |  |  |  |  | - http://www.doe.nv.gov/uploadedFiles/ndedoenvgov/content/home/DeclarationofEmergencyDirectiveSchools.pdf - http://www.doe.nv.gov/News__Media/Press_Releases/2020/Governor_Sisolak_Announces_Nevada_School_Buildings_will_not_Re-Open_During_the_2019-20_School_Year/ |
| Nevada | 3/17/20 |  |  | 🗸 |  |  | - http://gov.nv.gov/News/Emergency_Orders/2020/2020-03-18_-_COVID-19_Declaration_of_Emergency_Directive_002/ - http://gov.nv.gov/News/Press/2020/Governor_Sisolak_Announces_COVID-19_Risk_Mitigation_Initiatives/ |
| Nevada | 3/20/20 |  | 🗸 |  |  |  | - http://gov.nv.gov/News/Emergency_Orders/2020/2020-03-20_-_COVID-19_Declaration_of_Emergency_Directive_003/ - https://nvhealthresponse.nv.gov/wp-content/uploads/2020/03/3.20-Press-Release.pdf - https://nvhealthresponse.nv.gov/wp-content/uploads/2020/04/4.29-release.pdf |
| Nevada | 3/24/20 |  |  | 🗸 |  |  | - https://nvhealthresponse.nv.gov/wp-content/uploads/2020/03/03.24-PUBLIC-GATHERING-DIRECTIVE_.pdf - https://nvhealthresponse.nv.gov/wp-content/uploads/2020/04/4.29-release.pdf |
| Nevada | 4/1/20 |  |  |  | 🗸 |  | - https://nvhealthresponse.nv.gov/wp-content/uploads/2020/04/Declaration-of-Emergency-Directive-010-Stay-at-Home-3-31-20.pdf - https://nvhealthresponse.nv.gov/wp-content/uploads/2020/04/4.29-release.pdf |
| New Hampshire | 3/16/20 | 🗸 |  | 🗸 |  |  | - https://www.governor.nh.gov/news-media/orders-2020/documents/emergency-order-2.pdf - https://www.governor.nh.gov/news-media/orders-2020/documents/emergency-order-1.pdf - https://www.governor.nh.gov/news-media/emergency-orders/documents/emergency-order-26.pdf - https://www.governor.nh.gov/news-media/press-2020/documents/20200416-education-leaders.pdf |
| New Hampshire | 3/23/20 |  |  | 🗸 |  |  | - https://www.governor.nh.gov/news-media/emergency-orders/documents/emergency-order-16.pdf - https://www.governor.nh.gov/news-media/emergency-orders/documents/emergency-order-26.pdf |
| New Hampshire | 3/27/20 |  | 🗸 |  | 🗸 |  | - https://www.governor.nh.gov/news-media/emergency-orders/documents/emergency-order-17-1.pdf - https://www.governor.nh.gov/news-media/emergency-orders/documents/emergency-order-26.pdf - https://www.governor.nh.gov/news-media/emergency-orders/documents/emergency-order-40.pdf |
| New Hampshire | 4/7/20 |  |  |  | 🗸 |  | - <https://www.governor.nh.gov/news-media/emergency-orders/documents/emergency-order-28.pdf> |
| New Jersey | 3/16/20 |  |  | 🗸 |  |  | - <https://nj.gov/infobank/eo/056murphy/pdf/EO-104.pdf> |
| New Jersey | 3/18/20 | 🗸 |  |  |  |  | - https://nj.gov/governor/news/news/562020/approved/20200316c.shtml - https://nj.gov/infobank/eo/056murphy/pdf/EO-104.pdf |
| New Jersey | 3/21/20 | 🗸 | 🗸 | 🗸 | 🗸 |  | - <https://nj.gov/infobank/eo/056murphy/pdf/EO-107.pdf> |
| New Jersey | 4/1/20 |  |  | 🗸 |  |  | - <https://nj.gov/infobank/eo/056murphy/pdf/EO-110.pdf> |
| New Jersey | 4/7/20 |  |  | 🗸 |  |  | - <https://nj.gov/infobank/eo/056murphy/pdf/EO-118.pdf> |
| New Jersey | 4/10/20 |  |  | 🗸 |  |  | - <https://nj.gov/infobank/eo/056murphy/pdf/EO-122.pdf> |
| New Jersey | 4/13/20 |  |  | 🗸 |  |  | - <https://nj.gov/infobank/eo/056murphy/pdf/EO-125.pdf> |
| New Mexico | 3/16/20 | 🗸 |  | 🗸 |  |  | - https://www.governor.state.nm.us/wp-content/uploads/2020/03/Executive-Order-2020-005.pdf - https://www.governor.state.nm.us/2020/03/15/health-secretary-to-amend-public-health-order-adding-new-restrictions-to-public-gatherings/ - https://www.governor.state.nm.us/wp-content/uploads/2020/03/MLG_EO_2020_012.pdf |
| New Mexico | 3/19/20 |  |  | 🗸 |  |  | - https://www.governor.state.nm.us/2020/03/18/new-mexico-to-order-additional-closures-to-limit-spread-of-covid-19/ - https://www.governor.state.nm.us/wp-content/uploads/2020/03/SIGNED_UPDATED_DOH_PHO.pdf |
| New Mexico | 3/24/20 |  | 🗸 |  | 🗸 |  | - https://www.governor.state.nm.us/wp-content/uploads/2020/03/COVID-19-DOH-Order-fv.pdf - https://www.governor.state.nm.us/wp-content/uploads/2020/04/EO_2020_022.pdf - https://cv.nmhealth.org/2020/04/30/stay-at-home-order-extended/ |
| New Mexico | 3/27/20 |  |  |  |  | 🗸 | - https://www.governor.state.nm.us/wp-content/uploads/2020/03/MLG_EO_2020_013.pdf - https://www.governor.state.nm.us/wp-content/uploads/2020/03/MLG_EO_2020_013.pdf |
| New York | 3/16/20 |  |  | 🗸 |  |  | - <https://www.governor.ny.gov/news/no-2023-continuing-temporary-suspension-and-modification-laws-relating-disaster-emergency> |
| New York | 3/18/20 | 🗸 |  |  |  |  | - <https://www.governor.ny.gov/news/no-2024-continuing-temporary-suspension-and-modification-laws-relating-disaster-emergency> |
| New York | 3/20/20 |  | 🗸 |  |  |  | - <https://www.governor.ny.gov/news/no-2026-continuing-temporary-suspension-and-modification-laws-relating-disaster-emergency> |
| New York | 3/21/20 |  | 🗸 | 🗸 |  |  | - <https://www.governor.ny.gov/news/no-2027-continuing-temporary-suspension-and-modification-laws-relating-disaster-emergency> |
| New York | 3/22/20 |  | 🗸 |  | 🗸 |  | - https://www.governor.ny.gov/news/governor-cuomo-signs-new-york-state-pause-executive-order - https://www.governor.ny.gov/news/amid-ongoing-covid-19-pandemic-governor-cuomo-announces-nys-pause-extended-until-may-15 |
| North Carolina | 3/16/20 | 🗸 |  | 🗸 |  |  | - https://files.nc.gov/governor/documents/files/EO117-COVID-19-Prohibiting-Mass-Gathering-and-K12-School-Closure.pdf - https://files.nc.gov/governor/documents/files/EO120.pdf - https://files.nc.gov/governor/documents/files/EO138-Phase-1.pdf |
| North Carolina | 3/17/20 |  |  | 🗸 |  |  | - https://files.nc.gov/governor/documents/files/EO118.pdf - https://files.nc.gov/governor/documents/files/EO121-Stay-at-Home-Order-3.pdf - https://files.nc.gov/governor/documents/files/EO135-Extensions.pdf |
| North Carolina | 3/25/20 |  |  | 🗸 |  |  | - https://files.nc.gov/governor/documents/files/EO120.pdf - https://files.nc.gov/governor/documents/files/EO121-Stay-at-Home-Order-3.pdf - https://files.nc.gov/governor/documents/files/EO135-Extensions.pdf |
| North Carolina | 3/30/20 |  | 🗸 |  | 🗸 |  | - https://files.nc.gov/governor/documents/files/EO121-Stay-at-Home-Order-3.pdf - https://files.nc.gov/governor/documents/files/EO135-Extensions.pdf - https://files.nc.gov/governor/documents/files/EO138-Phase-1.pdf |
| North Dakota | 3/16/20 | 🗸 |  |  |  |  | - <https://www.governor.nd.gov/sites/www/files/documents/Executive%20Order%202020-04%20COVID-19%20school%20closing.pdf> |
| North Dakota | 3/20/20 |  |  | 🗸 |  |  | - <https://www.governor.nd.gov/sites/www/files/documents/executive-orders/Executive%20Order%202020-06.pdf> |
| North Dakota | 3/28/20 |  | 🗸 | 🗸 |  | 🗸 | - https://www.governor.nd.gov/sites/www/files/documents/executive-orders/Executive%20Order%202020-6.1%20expanded%20business%20closures.pdf - https://www.health.nd.gov/diseases-conditions/coronavirus/travel-quarantine-orders |
| North Dakota | 4/6/20 |  |  |  | 🗸 |  | - https://www.governor.nd.gov/sites/www/files/documents/executive-orders/Executive%20Order%202020-21%20-%20Quarantine%20of%20COVID-19%20positives%2C%20household%20contacts.pdf - https://www.governor.nd.gov/sites/www/files/documents/executive-orders/Executive%20Order%202020-21.1.pdf - https://www.governor.nd.gov/sites/www/files/documents/executive-orders/Executive%20Order%202020-21.2.pdf |
| Ohio | 3/15/20 |  |  | 🗸 |  |  | - [https://coronavirus.ohio.gov/wps/wcm/connect/gov/aa5aa123-c6c9-4e95-8a0d-bc77409c7296/Health+Director+Order+Limit+Food%2C+Alcohol+Sales+to+Carry+Out+Delivery+Only.pdf](https://coronavirus.ohio.gov/wps/wcm/connect/gov/aa5aa123-c6c9-4e95-8a0d-bc77409c7296/Health+Director+Order+Limit+Food%2C+Alcohol+Sales+to+Carry+Out+Delivery+Only.pdf?MOD=AJPERES&CONVERT_TO=url&CACHEID=ROOTWORKSPACE.Z18_M1HGGIK0N0JO00QO9DDDDM3000-aa5aa123-c6c9-4e95-8a0d-bc77409c7296-n3wHV26) |
| Ohio | 3/16/20 | 🗸 |  |  |  |  | - https://governor.ohio.gov/wps/portal/gov/governor/media/news-and-media/announces-school-closures - https://coronavirus.ohio.gov/wps/wcm/connect/gov/72a6497a-a816-4256-8555-cd5eab60278d/1923_001.pdf - https://content.govdelivery.com/attachments/OHOOD/2020/04/02/file_attachments/1418062/Signed%20Amended%20Director%27s%20Stay%20At%20Home%20Order.pdf - https://governor.ohio.gov/wps/portal/gov/governor/media/news-and-media/covid19-update-april-20-2020 |
| Ohio | 3/17/20 |  |  | 🗸 |  |  | - [https://coronavirus.ohio.gov/wps/wcm/connect/gov/dd504af3-ae2c-4d2e-b2bd-02c1a3beed89/Director%27s+Order-+Amended+Mass+Gathering+3.17.20+%281%29.pdf](https://coronavirus.ohio.gov/wps/wcm/connect/gov/dd504af3-ae2c-4d2e-b2bd-02c1a3beed89/Director%27s+Order-+Amended+Mass+Gathering+3.17.20+%281%29.pdf?MOD=AJPERES&CONVERT_TO=url&CACHEID=ROOTWORKSPACE.Z18_M1HGGIK0N0JO00QO9DDDDM3000-dd504af3-ae2c-4d2e-b2bd-02c1a3beed89-n3FI0mY) |
| Ohio | 3/21/20 |  |  | 🗸 |  |  | - [https://coronavirus.ohio.gov/wps/wcm/connect/gov/56be4a6a-3305-4697-97da-582d7b45da2f/Directors+Order+--+Internet+Cafes+03.21.20.pdf](https://coronavirus.ohio.gov/wps/wcm/connect/gov/56be4a6a-3305-4697-97da-582d7b45da2f/Directors+Order+--+Internet+Cafes+03.21.20.pdf?MOD=AJPERES&CONVERT_TO=url&CACHEID=ROOTWORKSPACE.Z18_M1HGGIK0N0JO00QO9DDDDM3000-56be4a6a-3305-4697-97da-582d7b45da2f-n42.Sg2) |
| Ohio | 3/23/20 |  | 🗸 |  | 🗸 |  | - https://governor.ohio.gov/static/DirectorsOrderStayAtHome.pdf - https://content.govdelivery.com/attachments/OHOOD/2020/04/02/file_attachments/1418062/Signed%20Amended%20Director%27s%20Stay%20At%20Home%20Order.pdf - https://coronavirus.ohio.gov/static/publicorders/Directors-Stay-Safe-Ohio-Order.pdf |
| Oklahoma | 3/17/20 | 🗸 |  |  |  |  | - https://twitter.com/oksde/status/1239662538289184768 - https://sde.ok.gov/sites/default/files/documents/files/20200325124831229.pdf |
| Oklahoma | 3/25/20 |  | 🗸 |  | 🗸 |  | - <https://www.sos.ok.gov/documents/executive/1919.pdf> |
| Oklahoma | 4/1/20 |  |  | 🗸 | 🗸 | 🗸 | - <https://www.sos.ok.gov/documents/executive/1926.pdf> |
| Oregon | 3/16/20 |  |  | 🗸 |  |  | - <https://www.oregon.gov/gov/Documents/executive_orders/eo_20-05.pdf> |
| Oregon | 3/16/20 | 🗸 |  |  |  |  | - https://www.oregon.gov/gov/Documents/executive_orders/eo_20-08.pdf - https://www.oregon.gov/gov/Documents/executive_orders/eo_20-20.pdf |
| Oregon | 3/17/20 |  |  | 🗸 |  |  | - https://www.oregon.gov/gov/Documents/executive_orders/eo_20-07.pdf - https://www.oregon.gov/gov/Documents/executive_orders/eo_20-14.pdf |
| Oregon | 3/23/20 |  | 🗸 |  | 🗸 |  | - <https://govsite-assets.s3.amazonaws.com/jkAULYKcSh6DoDF8wBM0_EO%2020-12.pdf> |
| Pennsylvania | 3/16/20 | 🗸 |  |  |  |  | - https://www.education.pa.gov/Schools/safeschools/emergencyplanning/COVID-19/messages/Pages/March-15,-2020.aspx - https://www.media.pa.gov/Pages/Education-Details.aspx?newsid=829 |
| Pennsylvania | 3/17/20 |  |  | 🗸 |  |  | - https://www.governor.pa.gov/wp-content/uploads/2020/03/20200319-TWW-COVID-19-business-closure-order.pdf - https://www.governor.pa.gov/newsroom/gov-wolf-puts-statewide-covid-19-mitigation-efforts-in-effect-stresses-need-for-every-pennsylvanian-to-take-action-to-stop-the-spread/ |
| Pennsylvania | 3/21/20 |  | 🗸 |  |  |  | - <https://www.governor.pa.gov/wp-content/uploads/2020/03/20200319-TWW-COVID-19-business-closure-order.pdf> |
| Pennsylvania | 4/1/20 |  |  |  | 🗸 |  | - https://www.governor.pa.gov/wp-content/uploads/2020/04/20200401-GOV-Statewide-Stay-at-Home-Order.pdf - https://www.governor.pa.gov/newsroom/gov-wolf-sec-of-health-extend-statewide-stay-at-home-order-until-may-8/ - https://www.governor.pa.gov/newsroom/gov-wolf-sec-of-health-take-actions-on-stay-at-home-orders-issue-yellow-phase-orders/ |
| Rhode Island | 3/17/20 |  |  | 🗸 |  |  | - <http://www.governor.ri.gov/documents/orders/Executive-Order-20-04.pdf> |
| Rhode Island | 3/23/20 | 🗸 |  | 🗸 |  |  | - https://www.ri.gov/press/view/37961 - http://www.governor.ri.gov/documents/orders/Executive-Order-20-09.pdf - https://governor.ri.gov/documents/orders/Executive-Order-20-23.pdf |
| Rhode Island | 3/24/20 |  |  | 🗸 |  | 🗸 | - <http://www.governor.ri.gov/documents/orders/Executive-Order-20-10.pdf> |
| Rhode Island | 3/30/20 |  | 🗸 | 🗸 | 🗸 |  | - http://www.governor.ri.gov/documents/orders/Executive-Order-20-14.pdf - http://governor.ri.gov/documents/orders/Executive-Order-20-23.pdf |
| South Carolina | 3/16/20 | 🗸 |  |  |  |  | - https://governor.sc.gov/sites/default/files/Documents/Executive-Orders/2020-03-15%20FILED%20Executive%20Order%20No.%202020-09%20-%20Closing%20Schools%20Cancelling%20Elections%20Other%20Provisions%20Due%20to%20COVID-19.pdf - https://governor.sc.gov/sites/default/files/Documents/Executive-Orders/2020-04-12%20eFILED%20Executive%20Order%20No.%202020-23%20-%20Third%20State%20of%20Emergency%20Due%20to%20COVID-19%20Pandemic.pdf - https://ed.sc.gov/newsroom/covid-19-coronavirus-and-south-carolina-schools/ |
| South Carolina | 3/18/20 |  |  | 🗸 |  |  | - https://governor.sc.gov/sites/default/files/Documents/Executive-Orders/2020-03-17%20eFILED%20Executive%20Order%20No.%202020-10%20-%20Directing%20Additional%20Emergency%20Measures%20Due%20to%20COVID-19.pdf https://governor.sc.gov/sites/default/files/Documents/Executive-Orders/2020-04-12%20eFILED%20Executive%20Order%20No.%202020-23%20-%20Third%20State%20of%20Emergency%20Due%20to%20COVID-19%20Pandemic.pdf |
| South Carolina | 3/23/20 |  |  | 🗸 |  |  | - <https://governor.sc.gov/sites/default/files/Documents/Executive-Orders/2020-03-23%20eFILED%20Executive%20Order%20No.%202020-13%20-%20Authorizing%20Law%20Enforcement%20to%20Preserve%20Public%20Health.pdf> |
| South Carolina | 3/27/20 |  |  |  |  | 🗸 | - https://governor.sc.gov/sites/default/files/Documents/Executive-Orders/2020-03-27%20eFILED%20Executive%20Order%20No.%202020-14%20-%20Self-Quarantine%20for%20Individuals%20from%20High-Risk%20Areas.pdf - https://governor.sc.gov/sites/default/files/Documents/Executive-Orders/2020-05-01%20eFILED%20Executive%20Order%20No.%202020-30%20-%20Rescinding%20Self-Quarantine%2C%20Lodging%2C%20%26%20Travel%20Restrictions%20for%20Individuals%20Entering%20S.C.%20from%20High-Risk%20Areas.pdf |
| South Carolina | 4/1/20 |  | 🗸 |  |  |  | - <https://governor.sc.gov/sites/default/files/Documents/Executive-Orders/2020-03-31%20eFILED%20Executive%20Order%20No.%202020-17%20-%20Closure%20of%20Non-Essential%20Businesses.pdf> |
| South Carolina | 4/7/20 |  |  | 🗸 | 🗸 |  | - <https://governor.sc.gov/sites/default/files/Documents/Executive-Orders/2020-04-06%20eFILED%20Executive%20Order%20No.%202020-21%20-%20Stay%20at%20Home%20or%20Work%20Order.pdf> |
| South Dakota | 3/15/20 |  | 🗸 |  |  |  | - https://sdsos.gov/general-information/executive-actions/executive-orders/assets/2020-05.PDF - https://sdsos.gov/general-information/executive-actions/executive-orders/assets/2020-09.PDF - https://sdsos.gov/general-information/executive-actions/executive-orders/assets/2020-22.PDF |
| South Dakota | 3/23/20 |  |  | 🗸 |  |  | - https://sdsos.gov/general-information/executive-actions/executive-orders/assets/2020-08.PDF - https://sdsos.gov/general-information/executive-actions/executive-orders/assets/2020-12.PDF - https://sdsos.gov/general-information/executive-actions/executive-orders/assets/2020-20.PDF |
| South Dakota | 3/24/20 | 🗸 |  |  |  |  | - https://doe.sd.gov/coronavirus/ - https://sdsos.gov/general-information/executive-actions/executive-orders/assets/2020-20.PDF |
| Tennessee | 3/20/20 | 🗸 |  |  |  |  | - https://www.tn.gov/education/news/2020/3/16/statement-from-commissioner-penny-schwinn-on-school-closures.html - https://www.tn.gov/education/news/2020/4/15/statement-from-commissioner-penny-schwinn-on-governor-s-recommendation-to-extend-school-closures--announcement-of-covid-19-child-wellbeing-task-force-.html |
| Tennessee | 3/23/20 |  |  | 🗸 |  |  | - <https://sos-tn-gov-files.tnsosfiles.com/forms/exec-order-lee17.pdf> |
| Tennessee | 3/23/20 |  |  | 🗸 |  |  | - https://sos-tn-gov-files.tnsosfiles.com/forms/exec-order-lee17.pdf - https://publications.tnsosfiles.com/pub/execorders/exec-orders-lee22.pdf - https://publications.tnsosfiles.com/pub/execorders/exec-orders-lee27.pdf |
| Tennessee | 3/31/20 |  | 🗸 |  | 🗸 |  | - https://publications.tnsosfiles.com/pub/execorders/exec-orders-lee22.pdf - https://publications.tnsosfiles.com/pub/execorders/exec-orders-lee27.pdf |
| Texas | 3/20/20 | 🗸 |  | 🗸 |  |  | - https://gov.texas.gov/uploads/files/press/EO-GA_08_COVID-19_preparedness_and_mitigation_FINAL_03-19-2020_1.pdf - https://tea.texas.gov/sites/default/files/Texas%20School%20Closures%20as%20of%20March%2015th%20at%201-45pm.pdf - https://gov.texas.gov/uploads/files/press/EO-GA-14_Statewide_Essential_Service_and_Activity_COVID-19_IMAGE_03-31-2020.pdf - https://gov.texas.gov/news/post/governor-abbott-issues-executive-order-to-safely-and-strategically-reopen-select-services-and-activities-in-texas |
| Texas | 3/28/20 |  |  |  |  | 🗸 | - <https://gov.texas.gov/uploads/files/press/EO-GA-11_airport_travel_reporting_COVID-19_IMAGE_03-26-2020.pdf> |
| Texas | 4/2/20 |  | 🗸 |  | 🗸 |  | - <https://gov.texas.gov/uploads/files/press/EO-GA-14_Statewide_Essential_Service_and_Activity_COVID-19_IMAGE_03-31-2020.pdf> |
| Utah | 3/16/20 | 🗸 |  |  |  |  | - https://governor.utah.gov/2020/03/13/gov-herbert-announces-two-week-dismissal-of-utahs-public-schools/ - https://coronavirus.utah.gov/governor-herbert-announces-extension-soft-closure-for-public-schools/ |
| Utah | 3/18/20 |  |  | 🗸 |  |  | - https://governor.utah.gov/2020/03/18/state-orders-restaurants-bars-to-suspend-dine-in-services-to-slow-spread-of-covid-19/ - https://drive.google.com/file/d/1PH2tbSfCvKjtw7LG1PgqhPclCaqiu-q6/view |
| Utah | 3/21/20 |  |  | 🗸 |  |  | - <https://drive.google.com/file/d/1Kp9RJEmiB_SCbWlEP4bo5e_c464SsCMW/view> |
| Utah | 4/1/20 |  |  |  | 🗸 |  | - <https://drive.google.com/file/d/1PH2tbSfCvKjtw7LG1PgqhPclCaqiu-q6/view> |
| Vermont | 3/13/20 |  |  | 🗸 |  |  | - <https://governor.vermont.gov/sites/scott/files/documents/EO%2001-20%20Declaration%20of%20State%20of%20Emergency%20in%20Response%20to%20COVID-19%20and%20National%20Guard%20Call-Out.pdf> |
| Vermont | 3/16/20 |  |  | 🗸 |  |  | - <https://governor.vermont.gov/content/addendum-1-executive-order-01-20> |
| Vermont | 3/17/20 |  |  | 🗸 |  |  | - https://governor.vermont.gov/content/addendum-2-executive-order-01-20 - https://governor.vermont.gov/sites/scott/files/documents/ADDENDUM%208%20TO%20EXECUTIVE%20ORDER%2001-20.pdf |
| Vermont | 3/18/20 | 🗸 |  |  |  |  | - https://education.vermont.gov/press-release/governor-scott-orders-orderly-closure-vermont-schools - https://governor.vermont.gov/sites/scott/files/documents/Directive%205%20-%20Continuity%20of%20Learning%20Planning%20_0.pdf |
| Vermont | 3/23/20 |  | 🗸 | 🗸 |  |  | - https://governor.vermont.gov/sites/scott/files/documents/ADDENDUM%209%20TO%20EXECUTIVE%20ORDER%2001-20.pdf - https://governor.vermont.gov/sites/scott/files/documents/ADDENDUM%209%20TO%20EXECUTIVE%20ORDER%2001-20.pdf |
| Vermont | 3/25/20 |  | 🗸 |  | 🗸 |  | - https://governor.vermont.gov/sites/scott/files/documents/ADDENDUM%206%20TO%20EXECUTIVE%20ORDER%2001-20.pdf - https://governor.vermont.gov/sites/scott/files/documents/ADDENDUM%209%20TO%20EXECUTIVE%20ORDER%2001-20.pdf |
| Vermont | 3/30/20 |  |  |  |  | 🗸 | - https://governor.vermont.gov/sites/scott/files/documents/ADDENDUM%207%20TO%20EXECUTIVE%20ORDER%2001-20.pdf - https://governor.vermont.gov/sites/scott/files/documents/ADDENDUM%209%20TO%20EXECUTIVE%20ORDER%2001-20.pdf |
| Virginia | 3/12/20 |  | 🗸 | 🗸 |  |  | - <https://www.governor.virginia.gov/newsroom/all-releases/2020/march/headline-853537-en.html> |
| Virginia | 3/16/20 | 🗸 |  | 🗸 |  |  | - https://www.governor.virginia.gov/newsroom/all-releases/2020/march/headline-854442-en.html - https://www.governor.virginia.gov/newsroom/all-releases/2020/march/headline-855292-en.html - https://www.governor.virginia.gov/media/governorvirginiagov/governor-of-virginia/pdf/Order-of-the-Governor-and-State-Health-Commissioner-Declaration-of-Public-Health-Emergency.pdf - https://www.governor.virginia.gov/media/governorvirginiagov/executive-actions/EO-55-Temporary-Stay-at-Home-Order-Due-to-Novel-Coronavirus-(COVID-19).pdf |
| Virginia | 3/24/20 |  |  | 🗸 |  |  | - https://www.governor.virginia.gov/media/governorvirginiagov/executive-actions/EO-53-Temporary-Restrictions-Due-To-Novel-Coronavirus-(COVID-19).pdf - https://www.governor.virginia.gov/media/governorvirginiagov/executive-actions/EO-55-Temporary-Stay-at-Home-Order-Due-to-Novel-Coronavirus-(COVID-19).pdf |
| Virginia | 3/30/20 |  | 🗸 |  | 🗸 |  | - <https://www.governor.virginia.gov/media/governorvirginiagov/executive-actions/EO-55-Temporary-Stay-at-Home-Order-Due-to-Novel-Coronavirus-(COVID-19).pdf> |
| Washington | 3/10/20 |  |  | 🗸 |  |  | - <https://www.governor.wa.gov/sites/default/files/proclamations/20-06%20Coronavirus%20%28tmp%29.pdf> |
| Washington | 3/13/20 |  |  | 🗸 |  |  | - <https://www.governor.wa.gov/sites/default/files/proclamations/20-11%20Coronavirus%20Gatherings%20Amendment%20%28tmp%29.pdf> |
| Washington | 3/16/20 |  |  | 🗸 |  |  | - https://www.governor.wa.gov/sites/default/files/proclamations/20-13%20Coronavirus%20Restaurants-Bars%20%28tmp%29.pdf - https://www.governor.wa.gov/sites/default/files/20-25.1%20-%20COVID-19%20-%20Stay%20Home%2C%20Stay%20Healthy%20Extension%20%28tmp%29.pdf |
| Washington | 3/17/20 | 🗸 |  |  |  |  | - https://www.governor.wa.gov/sites/default/files/proclamations/20-09%20Coronavirus%20Schools%20Amendment%20%28tmp%29.pdf - https://www.governor.wa.gov/sites/default/files/20-09.1%20-%20COVID-19%20School%20Closure%20Extension.pdf |
| Washington | 3/23/20 |  | 🗸 | 🗸 | 🗸 |  | - https://www.governor.wa.gov/sites/default/files/proclamations/20-25%20Coronovirus%20Stay%20Safe-Stay%20Healthy%20%28tmp%29%20%28002%29.pdf - https://www.governor.wa.gov/sites/default/files/20-25.1%20-%20COVID-19%20-%20Stay%20Home%2C%20Stay%20Healthy%20Extension%20%28tmp%29.pdf - https://www.governor.wa.gov/sites/default/files/20-25.3%20-%20COVID-19%20Stay%20Home%20Stay%20Healthy%20-%20Reopening%20%28tmp%29.pdf |
| West Virginia | 3/13/20 | 🗸 |  |  |  |  | - <https://governor.wv.gov/News/press-releases/2020/Pages/COVID19-UPDATE-Gov.-Justice-announces-closure-of-West-Virginia-schools.aspx> |
| West Virginia | 3/17/20 |  |  | 🗸 |  |  | - <https://governor.wv.gov/News/press-releases/2020/Pages/COVID-19-UPDATE-Gov.-Justice-holds-statewide-address-to-discuss-coronavirus-precautions.aspx> |
| West Virginia | 3/19/20 |  |  | 🗸 |  |  | - <https://governor.wv.gov/Documents/2020%20Executive%20Orders/Executive-Order-March-19-2020.pdf> |
| West Virginia | 3/20/20 |  |  | 🗸 |  |  | - <https://governor.wv.gov/Documents/2020%20Executive%20Orders/Executive-Order-March-20-2020-Barbers-Salons.pdf> |
| West Virginia | 3/24/20 |  | 🗸 |  | 🗸 |  | - <https://governor.wv.gov/Documents/2020%20Executive%20Orders/STAY-AT-HOME-ORDER-MARCH-23-2020.pdf> |
| West Virginia | 3/31/20 |  |  | 🗸 |  | 🗸 | - <https://governor.wv.gov/Documents/SGovernor%27s20033014470.pdf> |
| Wisconsin | 3/17/20 |  |  | 🗸 |  |  | - <https://content.govdelivery.com/attachments/WIGOV/2020/03/16/file_attachments/1402207/DHS%20Order%20Mass%20Gatherings%20of%2050%20or%20More.pdf> |
| Wisconsin | 3/17/20 |  |  | 🗸 |  |  | - https://evers.wi.gov/Documents/COVID19/UPDATEDOrder10People.pdf - https://content.govdelivery.com/attachments/WIGOV/2020/04/16/file_attachments/1428995/EMO28-SaferAtHome.pdf |
| Wisconsin | 3/18/20 | 🗸 |  |  |  |  | - https://evers.wi.gov/Documents/EO/SignedSchoolClosure.pdf - https://content.govdelivery.com/attachments/WIGOV/2020/04/16/file_attachments/1428995/EMO28-SaferAtHome.pdf - https://evers.wi.gov/Documents/COVID19/EMO28-SaferAtHome.pdf |
| Wisconsin | 3/25/20 |  | 🗸 |  | 🗸 |  | - https://content.govdelivery.com/attachments/WIGOV/2020/03/24/file_attachments/1409408/Health%20Order%20%2312%20Safer%20At%20Home.pdf - https://content.govdelivery.com/attachments/WIGOV/2020/04/16/file_attachments/1428995/EMO28-SaferAtHome.pdf |
| Wyoming | 3/19/20 | 🗸 |  | 🗸 |  |  | - https://health.wyo.gov/wp-content/uploads/2020/03/Statewide-Order-3.19.2020.pdf - https://drive.google.com/file/d/1509mzmlpF-EWGHEMX6izhE00wWKLXbGk/view - https://health.wyo.gov/wp-content/uploads/2020/04/Third-Continuation_Order1.pdf |
| Wyoming | 3/20/20 |  |  | 🗸 |  |  | - https://health.wyo.gov/wp-content/uploads/2020/03/March-20-gatherings-order.pdf - https://drive.google.com/file/d/1zGDHQQ5HFBbGPSSUVYykd9s7SZmpcSEn/view - https://governor.wyo.gov/media/news-releases/2020-news-releases/governor-gordon-authorizes-re-opening-of-gyms-personal-care-services-under |
| Wyoming | 3/25/20 |  |  | 🗸 |  |  | - https://health.wyo.gov/governor-state-health-officer-issue-third-closure-order/ - https://drive.google.com/file/d/1n8-RpUjeWZTm7UwuwSUR_tNARUf2zFpV/view - https://health.wyo.gov/wp-content/uploads/2020/04/Third-Continuation_Order1.pdf |
| Wyoming | 4/3/20 |  |  |  |  | 🗸 | - https://drive.google.com/file/d/1AXugf7iJuTaWjMYLpA1NS6i7SFJY_IWR/view - https://drive.google.com/file/d/1kEoIo4yHtYRoZlCSH3HQ9FW1FcTZkyc5/view |

*Notes: A, closure of schools; B, closure of workplaces; C, cancellation of public events; D, restrictions on internal movement; E, closure of state borders*

**S1 Text Table B.** Sensitivity analyses for the effect of implementation of any statewide social distancing measure on daily epidemic growth rate

| **Model** | **b** | **95% CI** | ***P*-value** |
| --- | --- | --- | --- |
| Unadjusted estimate with 3-day incubation period * | -0.009 | -0.014, -0.004 | <0.001 |
| Adjusted for population density and day of week | -0.008 | -0.013, -0.004 | <0.001 |
| Unadjusted estimate, among states with <100 cases at the time the first statewide social distancing measure was implemented | -0.002 | -0.013, 0.010 | 0.79 |
| Unadjusted estimate, among states with ≥100 cases at the time the first statewide social distancing measure was implemented | -0.009 | -0.015, 0.003 | 0.005 |

*Notes: b, estimated regression coefficient; CI, confidence interval. Each row corresponds to a separate regression model fitted to the data. The primary analysis (*) is based on a regression model that specifies a 3-day incubation period and that includes 3 variables: time in days, implementation period, and a time-by-implementation period product term.*

**S1 Text Table C.** Sensitivity analyses for the effect of implementation of statewide restriction on internal movement on daily epidemic growth rate

| **Model** | **b** | **95% CI** | ***P*-value** |
| --- | --- | --- | --- |
| Unadjusted estimate with 3-day incubation period * | 0.001 | 0.000, 0.003 | 0.14 |
| Adjusted for population density and day of week | 0.001 | 0.001, 0.003 | 0.14 |
| Unadjusted estimate, among states with <500 cases at the time the statewide restriction on internal movement was implemented | 0.000 | -0.003, 0.003 | 0.99 |
| Unadjusted estimate, among states with ≥500 cases at the time the statewide restriction on internal movement was implemented | 0.004 | 0.002, 0.006 | 0.001 |

*Notes: b, estimated regression coefficient; CI, confidence interval. Each row corresponds to a separate regression model fitted to the data. The primary analysis (*) is based on a regression model that specifies a 3-day incubation period and that includes 3 variables: time in days, implementation period, and a time-by-implementation period product term.*

**S1 Text Table D.** Sensitivity analyses for the effect of implementation of any statewide social distancing measure on daily epidemic growth rate, with assumed incubation periods of varying duration

| **Assumed incubation period** | **b** | **95% CI** | **P-value** |
| --- | --- | --- | --- |
| -2-day incubation period | -0.008 | -0.019, 0.003 | 0.140 |
| -1-day incubation period | -0.010 | -0.019, 0.001 | 0.025 |
| 0-day incubation period | -0.012 | -0.019, -0.004 | 0.002 |
| 1-day incubation period | -0.012 | -0.018, -0.006 | <0.001 |
| 2-day incubation period | -0.011 | -0.017, -0.006 | <0.001 |
| 3-day incubation period * | -0.009 | -0.014, -0.004 | <0.001 |
| 4-day incubation period | -0.007 | -0.012, -0.003 | <0.001 |
| 5-day incubation period | -0.001 | -0.004, -0.001 | 0.21 |
| 6-day incubation period | -0.005 | -0.008, -0.001 | 0.005 |
| 7-day incubation period | -0.004 | -0.008, 0.001 | 0.011 |
| 8-day incubation period | 0.002 | 0.005, 0.004 | 0.019 |

*Notes: b, estimated regression coefficient; CI, confidence interval. Each row corresponds to a separate regression model fitted to the data. The primary analysis (*) is based on a regression model that specifies a 3-day incubation period and that includes 3 variables: time in days, implementation period, and a time-by-implementation period product term.*

**S1 Text Fig A.** Timeline of implementation of first statewide social distancing measures and statewide restrictions on internal movement. Figure design adapted from Adolph and colleagues [2].

**S1 Text Fig B**. Changes in mean daily case growth rate before versus after implementation of the first statewide social distancing measures, in states that implemented such measures versus daily case growth in states that did not implement such measures


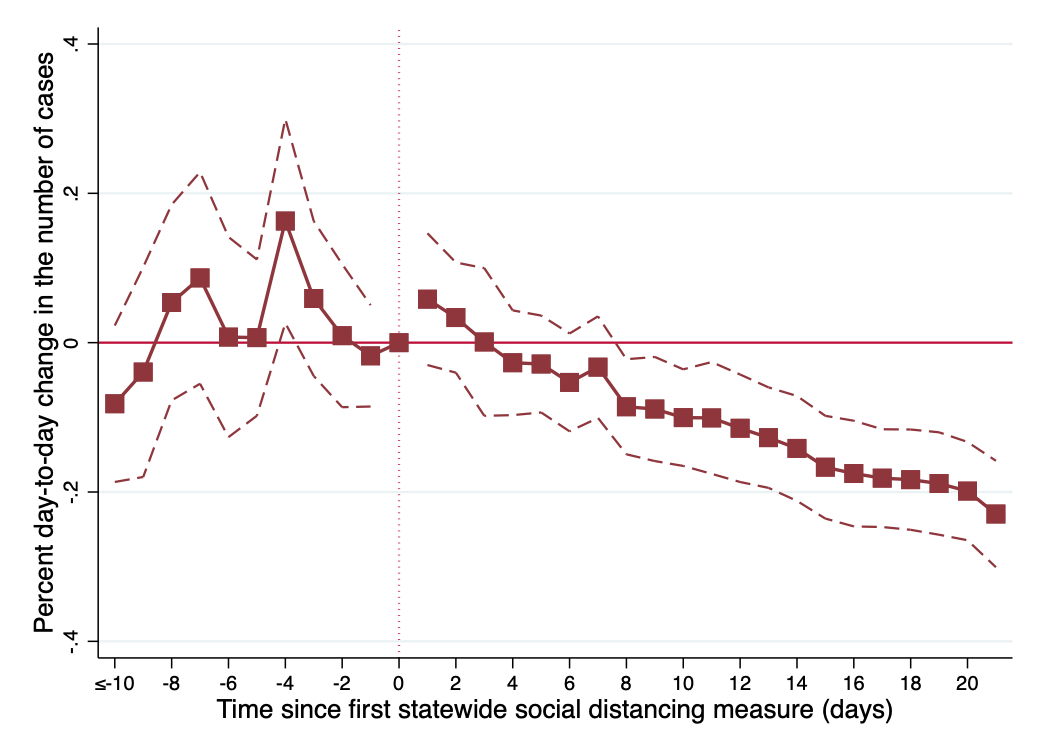


*Note: Dashed lines indicate 95% confidence intervals*

**S1 Text Fig C**. Changes in mean daily COVID-19-attributed deaths before versus after implementation of the first statewide social distancing measures, in states that implemented such measures versus daily COVID-19-attributed deaths in states that did not implement such measures


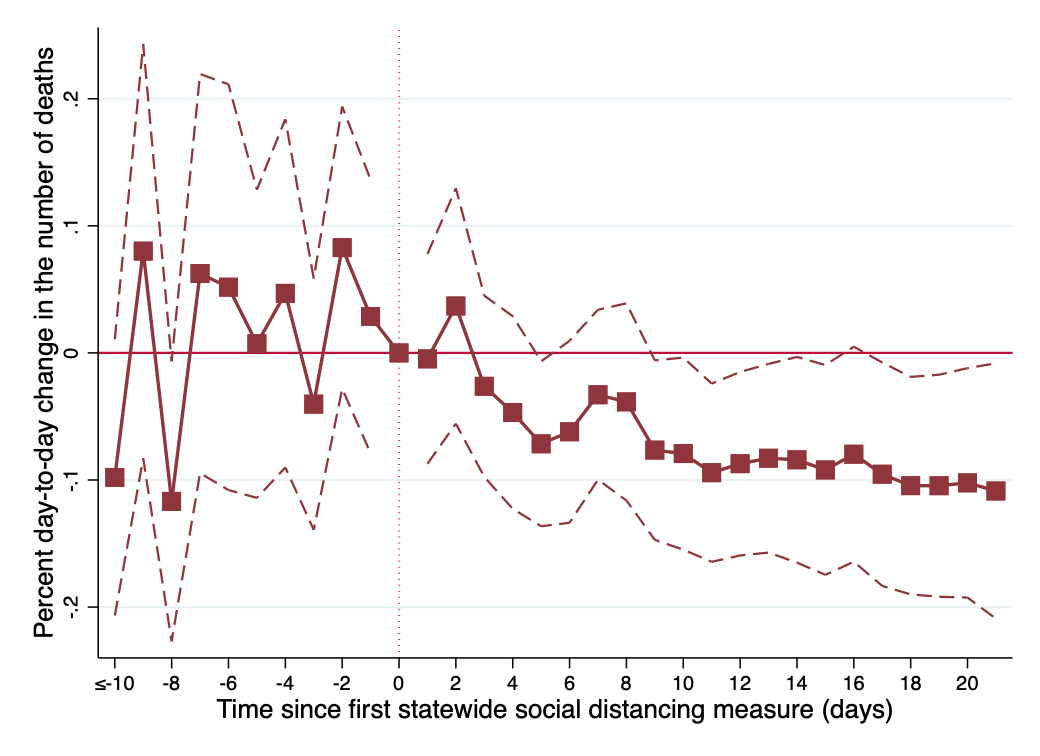


*Note: Dashed lines indicate 95% confidence intervals*

Supplement References

1. Petherick A, Hale T, Phillips T, Webster S. Variation in government responses to COVID-19 [preprint]. Blavatnik School Working Paper. Oxford: Blavatnik School of Government, University of Oxford, 2020. Available at: <https://www.bsg.ox.ac.uk/research/publications/variation-government-responses-covid-19>. Accessed 2 Jun 2020.

2. Adolph C, Amano K, Bang-Jensen B, Fullman N, Wilkerson J. Pandemic politics: timing state-level social distancing responses to COVID-19 [preprint]. medRxiv. 2020: Epub 31 Mar 2020. doi:10.1101/2020.03.30.20046326.

3. Lee VJ, Aguilera X, Heymann D, Wilder-Smith A, Lancet Infectious Diseases C. Preparedness for emerging epidemic threats: a Lancet Infectious Diseases Commission. Lancet Infect Dis. 2020;20(1):17-9.

4. Grassly NC, Fraser C. Mathematical models of infectious disease transmission. Nature reviews Microbiology. 2008;6(6):477-87.

5. Lewis MD, Pavlin JA, Mansfield JL, O'Brien S, Boomsma LG, Elbert Y, et al. Disease outbreak detection system using syndromic data in the greater Washington DC area. Am J Prev Med. 2002;23(3):180-6.

6. Buckingham-Jeffery E, Morbey R, House T, Elliot AJ, Harcourt S, Smith GE. Correcting for day of the week and public holiday effects: improving a national daily syndromic surveillance service for detecting public health threats. BMC Public Health. 2017;17(1):477.

7. Hatchett RJ, Mecher CE, Lipsitch M. Public health interventions and epidemic intensity during the 1918 influenza pandemic. Proc Natl Acad Sci U S A. 2007;104(18):7582-7.

8. Lauer SA, Grantz KH, Bi Q, Jones FK, Zheng Q, Meredith HR, et al. The incubation period of coronavirus disease 2019 (COVID-19) from publicly reported confirmed cases: estimation and application. Ann Intern Med. 2020;172(9):577-82.

9. Backer JA, Klinkenberg D, Wallinga J. Incubation period of 2019 novel coronavirus (2019-nCoV) infections among travellers from Wuhan, China, 20-28 January 2020. Euro Surveill. 2020;25(5).

10. Brzezinski A, Deiana G, Kecht V, Van Dijcke D. The COVID-19 pandemic: government vs. community action across the United States [preprint]. INET Oxford Working Paper No. 2020-06. Oxford: Institute for New Economic Thinking at the Oxford Martin School, University of Oxford, 2020. Available at: <https://www.inet.ox.ac.uk/publications/no-2020-06-the-covid-19-pandemic-government-vs-community-action-across-the-united-states/>. Accessed 2 Jun 2020.

11. Sullivan D, von Wachter T. Job displacement and mortality: an analysis using administrative data. Q J Econ. 2009;124(3):1265-306.

12. Jacobson L, LaLonde RJ, Sullivan DG. Earnings losses of displaced workers. Am Econ Rev. 1993;83(4):685-709.

13. Goodman-Bacon A. Difference-in-differences with variation in treatment timing [preprint]. NBER Working Paper No. 25018. Cambridge: National Bureau of Economic Research, 2018. Available at: <https://www.nber.org/papers/w25018>. Accessed 2 Jun 2020.

14. Bertrand M, Duflo E, Mullainathan S. How much should we trust differences-in-differences estimates? Q J Econ. 2004;119(1):249-75.

15. Siedner MJ, Harling G, Reynolds Z, Gilbert RF, Venkataramani A, Tsai AC. Social distancing to slow the U.S. COVID-19 epidemic: an interrupted time-series analysis [preprint]. medRxiv. 2020: Epub 8 Apr 2020. doi:10.1101/2020.04.03.20052373.
